# Analysis of the current situation and influencing factors of internal perceptions in patients with chronic heart failure at the county level

**DOI:** 10.3389/fpsyg.2025.1618068

**Published:** 2025-07-17

**Authors:** Ming Yu, Jiahong Ge, Lili Jiang, Rong Tang, Jing Ji, Dandan Gu, Suping Cai, Dawei Zhu, Jiaojian Shi, Shuangfeng Chen, Wenjuan Zhou, Ronghui Geng, Lingling Jiang

**Affiliations:** ^1^Department of Nursing, Affiliated Rudong Hospital of Xinglin College, Nantong University, Nantong, Jiangsu, China; ^2^Department of Medical, Affiliated Rudong Hospital of Xinglin College, Nantong University, Nantong, Jiangsu, China; ^3^Department of Medical, Tongzhou District Hospital of Traditional Chinese Medicine, Nantong, Jiangsu, China; ^4^Department of Research and Education, Affiliated Rudong Hospital of Xinglin College, Nantong University, Nantong, Jiangsu, China

**Keywords:** chronic heart failure, internal perception, influencing factors, social support, psychological state

## Abstract

**Aim:**

The objective of this study is to investigate interoceptive awareness—defined as the ability to perceive and interpret bodily signals—in individuals diagnosed with chronic heart failure, along with the factors that may influence this awareness. This research aims to establish a theoretical foundation for the development of psychoeducational programs focused on interoception, such as mindfulness-based symptom awareness training, and digital health interventions specifically designed for patients with chronic heart failure (CHF).

**Methods:**

A total of 406 patients diagnosed with congestive heart failure were selected as research subjects through convenience sampling from the cardiology departments of six county hospitals in Nantong City, during the period from January 2025 to March 2025. Cross-sectional surveys were conducted using various instruments, including general information questionnaires, the Multidimensional Internal Perception Assessment Questionnaire, the Social Support Scale, the Self-Rating Depression Scale, and the Memorial Heart Failure Symptom Assessment. To identify the factors influencing internal perception among CHF patients, both univariate and multivariate linear regression analyses were employed.

**Results:**

The overall score of the Multidimensional Assessment of Interoceptive Awareness 2C (MAIA-2C) for individuals diagnosed with congestive heart failure was recorded at 108.44 ± 15.93, within a scale range of 31–155, indicating a moderate-to-high degree of internal perception. A multivariate linear regression analysis identified several independent factors that significantly influence internal perception among CHF patients. These factors include place of residence, the frequency of hospitalizations due to heart failure in the past year, body mass index, levels of depression, availability of social support, and symptom burden.

**Conclusion:**

The internal perception ability of patients with congestive heart failure is influenced by various factors, including their residential environment, the number of hospitalizations related to heart failure in the past year, body mass index, levels of depression, the availability of social support, and symptom burden. In clinical settings, it is essential to consider the level of internal perception among patients. Furthermore, tailored intervention strategies should be developed, taking into account these influencing factors, in order to improve patients’ self-management skills and overall quality of life.

## Introduction

Individuals diagnosed with chronic heart failure (CHF) are at an elevated risk of readmission to healthcare facilities and face significant challenges that affect their overall quality of life (Spethmann et al., 2024). The 2024 Guidelines for the Diagnosis and Treatment of Heart Failure emphasize the importance of enhancing symptom awareness and self-management strategies, as these factors can lead to improved clinical outcomes and a more favorable prognosis (Beghini et al., 2025).

Interoceptive awareness, defined as the ability to perceive internal physiological states, plays a crucial role in mediating the relationship between somatic signals and self-regulatory behaviors (Craig, 2002). A lack of this awareness is linked to impaired emotional processing and maladaptive health responses during public health emergencies (Ceccato et al., 2021; Ceccato et al., 2023). In individuals with CHF, diminished interoceptive accuracy may exacerbate the misinterpretation of symptoms, as understood through the precognitive framework of embodied self-awareness (Khalsa et al., 2018).

In recent years, the biopsychosocial medical model has undergone significant advancements, prompting greater interest among academics in the fundamental concept of interoception (Chiesa et al., 2025). Interoception is defined as an individual’s ability to perceive, integrate, and interpret internal physiological signals. Empirical research has shown that continuous monitoring of internal perception in patients with chronic illnesses can significantly improve the efficacy of self-health management, thereby optimizing clinical outcomes and enhancing quality of life (Mayer-Benarous et al., 2025). Nevertheless, there is a significant gap in research regarding endometrial health among patients with CHF in China.

Using Rodgers’ evolutionary concept analysis framework, it is evident that the intrinsic perception mechanisms of patients with chronic illnesses exhibit significant heterogeneity (Hewko et al., 2025). The formation and evolution of these mechanisms are influenced by various interaction pathways, including biological factors (age, gender, and the presence of comorbid conditions), disease characteristics (the New York Heart Association [NYHA] classification and left ventricular ejection fraction [LVEF]), psychosocial factors (levels of anxiety, depression, and disease-related cognition), and the social support system (Vest et al., 2024). In this context, the current study employs a cross-sectional design to assess the intrinsic perception levels of patients diagnosed with CHF using standardized measurement scales. This study will systematically identify and analyze the primary predictors and mechanisms involved, thereby providing a scientific foundation for the development of precision nursing intervention programs specifically designed to address intrinsic perceptions. Ultimately, this research aims to promote a paradigm shift for CHF patients from a model of “passive treatment” to one of “active health management.”

## Methods

### Study design, sample, and procedure

A total of 406 patients with CHF were selected through convenience sampling from the cardiovascular departments of six county-level hospitals in Nantong from January 2025 to March 2025. The inclusion criteria were as follows: (1) meeting the diagnostic criteria for CHF (Spaderna et al., 2024), (2) age ≥ 18 years, and (3) the ability to operate a smartphone. The exclusion criteria included: (1) the presence of serious diseases or complications, such as malignant tumors or severe infections, (2) hearing and vision impairments that hinder communication, and (3) a history of mental illness or significant cognitive impairments that affect comprehension, expression, memory, and orientation. The initial sample size was estimated to be 5–10 times the number of variables (31 variables), resulting in a range of 186 to 372 cases.

To account for potential dropouts and ensure a robust multivariate analysis, we enrolled 406 participants. With this sample size, the study achieved 90% power to detect small-to-moderate effect sizes (Cohen’s *f*^2^ ≥ 0.08) in multivariate regression at *α* = 0.05, as calculated using G*Power 3.1. During the model refinement process, only variables with significant univariate associations (*p* < 0.05) were retained, resulting in six predictors for the final regression model. All respondents provided informed consent.

This study was approved by the Ethics Committee of Rudong County People’s Hospital (No. 54 [2024]), and all respondents provided informed consent and participated voluntarily.

In this study, we investigated the current status of intrinsic perception, social support, depression, and symptom burden in patients with CHF and analyzed the factors influencing intrinsic perception. Prior to data collection, researchers received standardized training to ensure consistency and reliability. During the data collection process, the investigator clearly explained the study’s purpose and the questionnaire completion method to the patients. The questionnaire was distributed only after obtaining their consent. Patient general information was collected through a review of medical records and in-person interviews, while disease history and biochemical indicators were obtained by examining the medical records. During the questionnaire completion process, the researcher utilized a standardized guide to address any questions. The questionnaires were distributed on-site, and completed before being checked for accuracy, with any omissions promptly addressed. Ultimately, a total of 410 questionnaires were distributed, and 406 valid responses were collected, yielding a recovery rate of 99.02%.

### Questionnaires

#### General information questionnaire

The researchers developed the questionnaire after reviewing the relevant literature, which included demographic information about the patients and pertinent data regarding their diseases. The questionnaire consists of a total of 17 items.

#### Multidimensional assessment of interoceptive awareness (MAIA-2C)

MAIA-2C refers to the Chinese adaptation of the multidimensional internal sensory assessment questionnaire, revised by Teng et al. (2022). This adaptation is based on the MAIA-2 scale developed by Mehling et al. (2018). This universal tool is designed to measure internal perception and has been used with 627 young individuals at Zhejiang University. The scale consists of a total of 31 items across 7 dimensions, including physical attention and emotional awareness. It utilizes a 6-point Likert scale, with total scores ranging from 31 to 155 points; a higher score indicates a stronger internal perception ability. In this study, the Cronbach’s *α* coefficient was found to be 0.890.

### Social support rate scale (SSRS)

The SSRS scale was revised by Shuiyuan Xiao (Jiang Y. et al., 2025) and includes three dimensions: objective support, subjective support, and utilization of support. The scale comprises a total of 10 items, with scores ranging from 12 to 66 points. A score of 22 or below indicates low social support, scores between 23 and 44 indicate medium social support, and scores of 45 or higher indicate high social support, with higher scores reflecting greater levels of social support. The Cronbach’s *α* coefficient for the scale was 0.78, and the test–retest reliability was 0.92. In this study, the Cronbach’s α coefficient for the scale was 0.806, while the overall Cronbach’s α coefficient for this study was 0.910.

### Self-rating depression scale (SDS)

Compiled by Zung (Cui et al., 2025), this scale consists of 20 items, including 10 forward-scoring items and 10 reverse-scoring items Each item is categorized into four levels based on the frequency of symptoms: Positive-scoring criteria are as follows: “No or occasional,” and are assigned scores of 1, 2, 3, and 4, respectively. The reverse-scoring criteria are as follows: “No or occasional,” and are scored as 4, 3, 2, and 1, respectively. The total score for each item is multiplied by 1.25 and then rounded to the nearest whole number. A standard score of ≥53 indicates the presence of depressive symptoms, with scores of 53–62 indicating mild depression, 63–72 indicating moderate depression, and scores greater than 72 indicating severe depression. In this study, the Cronbach’s *α* coefficient for the scale was 0.890.

### Memorial symptom assessment scale – heart failure (MASAS-HF)

MASAS-HF was developed by the American scholar Zambroski (Jiang Y. et al., 2025) in 2005, and it was later adapted by Guo Jinyu in 2014 to assess the symptomatic experiences and feelings of patients with heart failure. The scale comprises three subscales and a total of 32 symptoms: the physiological symptom sub-scale (21 symptoms), the psychological symptom subscale (6 symptoms), and the heart failure symptom subscale (5 symptoms). Each symptom is evaluated based on four key aspects: presence, frequency of occurrence (measured on a 4-point Likert scale) ranging from “rare” to “almost all the time severity (also on a 4-point Likert scale) from “mild” to “very severe distress (assessed on a 5-point Likert scale) from “No” to “very severe to 384 points, with higher scores indicating more severe symptoms. The Cronbach’s *α* coefficient for the total scale and its scaleless measure ranges from 0.807 to 0.946, demonstrating strong reliability. This scale has been widely utilized in studying heart failure symptoms both domestically and internationally. In this study, the total scale and scaleless measures were assessed, with the Cronbach’s α coefficients ranging from 0.845 to 0.932.

### Statistical analysis

The database was created by two individuals using EpiData 3.1 software, while the Statistical Package for the Social Sciences (SPSS) version 26.0 software was utilized for data analysis. Normally distributed continuous data were expressed as means with standard deviations, whereas non-normally distributed continuous data were represented as medians with interquartile ranges. For group comparisons, either the *t*-test for independent samples or the Mann–Whitney *U* test was employed. Categorical data were presented as frequencies and percentages, with the chi-squared test used for group comparisons. Multivariate analysis was conducted using stepwise regression analysis. Non-normally distributed data were analyzed with the Mann–Whitney *U* test. Multicollinearity was assessed using the inflation factor (VIF), ensuring that variables met the criterion of < 5. Non-linear relationships were tested and examined, including quadratic terms (e.g., BMI^2^) in sensitivity analyses. A *p*-value of less than 0.05 was considered statistically significant.

## Results

### General information and the intrinsic perception level of the respondents

The age of the survey respondents ranged from 46 to 84 (70.76 ± 9.43) years, and other general details are presented in Table 1. The total score of MAIA-2C was (108.44 ± 15.93), and the scores of each dimension were as follows: self-regulation (3.64 ± 0.58) and trust (3.62 ± 0.51). The lowest scores were observed for distraction (3.26 ± 0.60) and no worry (3.25 ± 0.63) (Table 2).

**Table 1:** General information of respondents and results of univariate analysis of nutritional status (*n* = 406).

Subject	*N* (%)	MAIA-2C ($\bar{x}\;$± *S*)	ANOVA	*p*
Age (years)			1.552^1)^	0.121
<60	206	109.65 ± 12.08		
>60	200	107.2 ± 19.05		
Gender			0.522^1)^	0.602
Male	201	108.86 ± 15.47		
Female	205	108.03 ± 16.39		
Education			3.868^2)^	**0.01 < 0.05**
Elementary school and below	174	105.99 ± 14.32		
Junior high school	91	107.92 ± 15.45		
High school	81	110.7 ± 18.77		
College degree or above	60	113.27 ± 15.81		
Occupation			2.390^2)^	0.050
Clerks/workers	123	110.76 ± 15.87		
Individual	5	113.2 ± 8.44		
Farmer	46	107.15 ± 17.26		
Retired	135	109.35 ± 16.79		
Unemployed	97	104.6 ± 13.76		
Marital status			13.932^2)^	**<0.001**
Unmarried	6	111.67 ± 5.68		
Married	376	109.41 ± 15.18		
Divorced/widowed	24	92.33 ± 20.38		
Per capita monthly household income (RMB)			−2.768^1)^	**0.006 < 0.05**
<3,000	251	106.73 ± 14.67		
≥3,000	155	111.2 ± 17.48		
Place of residence			3.225^1)^	**0.001 < 0.05**
City/county seat	153	111.68 ± 16.99		
Town/township/village	253	106.48 ± 14.95		
Residency style			−0.476^1)^	0.634
Living alone	195	104.87 ± 14.67		
Cohabitation with another person	211	111.73 ± 16.36		
Family history of cardiovascular disease			−1.378^1)^	0.169
Yes	249	107.57 ± 14.96		
No	157	109.81 ± 17.32		
History of smoking			0.470^1)^	0.639
Yes	193	108.83 ± 15.25		
No	213	108.08 ± 16.55		
History of alcohol consumption			1.976^1)^	0.050
Yes	90	111.36 ± 18.15		
No	316	107.61 ± 15.17		
Total number of hospitalizations for heart failure in the last 1 year			7.871^2)^	**<0.001**
1–10	344	109.03 ± 16.18		
11–15	52	108.23 ± 10.73		
>15	10	89.1 ± 19.13		
NYHA grading			3.099^2)^	0.050
II	89	111.97 ± 16.16		
III	221	107.01 ± 17.44		
IV	96	108.45 ± 10.93		
Number of comorbidities			4.278^2)^	**0.005 < 0.05**
1	105	112.99 ± 15.38		
2	252	107.19 ± 15		
3	47	104.85 ± 20		
4	2	110.5 ± 12.02		
BMI			4.921^2)^	**0.008 < 0.05**
<18.5	22	98.27 ± 16.18		
18.5–24.9	282	108.81 ± 14.11		
>24.9	102	109.6 ± 19.62		
LVEF (%)			2.935^1)^	**0.004 < 0.05**
>50	140	111.61 ± 17.45		
<50	266	106.77 ± 14.84		
Weak			−2.777^1)^	**0.006 < 0.05**
Yes	245	106.67 ± 14.47		
No	161	111.12 ± 17.63		

MAIA-2C, Multidimensional Assessment of Interoceptive Awareness; ANOVA, analysis of variance; BMI, body mass index; LVEF, left ventricular ejection fraction; RMB, renminbi. 1) *t*; 2) *F*. Bold values indicate statistical significance (*p* < 0.05).

**Table 2:** MAIA-2C scale scores of CHF patients (*n* = 406).

Subject	Number of entries	Score range	Minimum score	Highest score	Total score ($\bar{x\;}\;$ ± *S*)	Entries are divided equally ($\bar{x\;}\;$± *S*)
Total score	31	31–155	48	155	108.44 ± 15.93	3.5 ± 0.51
No distractions	5	25	8	25	16.3 ± 3	3.26 ± 0.6
No worry	5	25	5	25	16.25 ± 3.17	3.25 ± 0.63
Pay attention to the adjustment	3	15	3	15	10.81 ± 1.73	3.6 ± 0.58
Emotional awareness	4	20	4	20	14.41 ± 2.26	3.6 ± 0.56
Self-regulating	4	20	4	20	14.54 ± 2.33	3.64 ± 0.58
Body listening	4	20	4	20	14.43 ± 2.19	3.61 ± 0.55
Trust	6	30	12	30	21.69 ± 3.04	3.62 ± 0.51

CHF, chronic heart failure; MAIA-2C, Multidimensional Assessment of Interoceptive Awareness.

### Univariate analysis of factors influencing intrinsic perception in CHD patients

There were statistically significant differences in MAIA-2C scores among CHF patients with varying education levels, marital status, per capita monthly family income, place of residence, number of hospitalizations for heart failure in the past year, number of comorbidities, BMI, LVEF, and frailty (all *p* < 0.05). (Table 1).

### Multivariate linear regression analysis

The total score of the MAIA-2C was utilized as the dependent variable. The meaningful variables identified in the univariate analysis were incorporated into the regression model, with the original scores for depression, social support, and symptom burden being substituted. The independent variables were assigned as detailed in Table 3. Stepwise regression analysis identified the following variables as independent predictors of MAIA-2C scores: place of residence, number of hospitalizations, body mass index (BMI), depression, social support, and symptom burden (see Table 4). To further investigate the non-linear relationship between BMI and internal perception, a quadratic term (BMI^2^) was added to the model. The significant coefficient for BMI^2^ (*β* = −0.05, SE = 0.02, and *p*s = 0.04 *p* = 0.04)—a U-shaped relationship, which suggests that low and high BMI values were associated with reduced perception. Stratified analysis based on BMI categories further supported the threshold effect (Table 5).

**Table 3:** Assignment of independent variables.

Subject	Assignment method
Education	Elementary school and below = 0, junior high school = 1, high school = 2, college degree or above = 3
Marital status	Unmarried (Z1 = 0, Z2 = 0), married (Z1 = 1, Z2 = 0), Divorced/widowed (Z1 = 0, Z2 = 1)
Monthly income per capita	<3,000 = 0, ≥3,000 = 1
Place of residence	City/county seat = 0, Town/township/village = 1
Heart failure in the last 1 year Total number of hospitalizations	1–10 = 1, 11–15 = 2, >15 = 3
Number of types of comorbidities	1 = 0, 2 = 1, 3 = 2, 4 = 3
BMI	<18.5 = 0, 18.5–24.9 = 1, >24.9 = 2
LVEF	>50 = 0, <50 = 1
Weak	Yes = 0, No = 1

BMI, body mass index; LVEF, left ventricular ejection fraction.

**Table 4:** Results of multivariate regression analysis of factors influencing intrinsic perceptions in CHF patients.

Subject	Partial regression coefficient	SE	Standard regression coefficients	Cohen’s *f*^2^	*t*	*p*
(Constant)	109.855	5.455			20.139	<0.001
Place of residence	−2.316	0.713	−0.071	0.027	−3.251	0.001 < 0.05
Number of hospitalizations for heart failure in the last 1 year	−2.073	0.632	−0.058	0.027	−3.283	0.001 < 0.05
BMI	−1.475	0.655	−0.048	0.013	−2.251	0.025 < 0.05
SDS	−0.961	0.043	−0.586	1.286	−22.561	<0.001
SSRS	1.038	0.07	0.392	0.551	14.859	<0.001
MASAS-HF	−0.016	0.006	−0.048	0.018	−2.513	0.012 < 0.05

*R*^2^ = 0.917, adjusted *R*^2^ = 0.916, *F* = 735.270, *p* < 0.001. Effect size standards: *f*^2^ ≥ 0.02 (low); *f*^2^ ≥ 0.15 (medium); and *f*^2^ ≥ 0.35 (high). SE, standard error; BMI, body mass index; SDS, Self-Rating Depression Scale; SSRS, Social Support Rate Scale; MASAS-HF, Memorial Symptom Assessment Scale – Heart Failure.

**Table 5:** Stratified analysis of BMI effects on MAIA-2C scores (*N* = 406).

BMI category	*β*	SE	Cohen’s *f*^2^	*p*
Normal (18.5 ~ 24.9)	−0.85	0.71	0.01	0.18
Overweight/Obese (≥24.9)	−2.10	0.68	0.09	0.01

BMI, body mass index; SE, standard error.

## Discussion

### Status of internal perception in CHF patients

In this study, we analyzed the internal perception abilities of 406 patients with CHF across six county hospitals. The average MAIA-2C score of the patients was 3.50 ± 0.51, indicating an upper-middle level of awareness. This suggests that the majority of patients possess a fundamental ability to recognize, integrate, and interpret physiological signals, enabling them to identify typical symptoms associated with changes in cardiac function. However, within the sub-dimensions of internal perception, the scores for “no distraction” (3.26 ± 0.60) and “no worry” (3.25 ± 0.63) were the lowest. This reflects the challenges these patients face in maintaining focus and managing anxiety in response to physical discomfort, which may be linked to their disease state, prolonged treatment experiences, and psychological stress.

In light of the prolonged course of CHF and the recurrence of symptoms, patients must contend with practical challenges, such as decreased activity tolerance and a diminished quality of life, over an extended period (Whitfield et al., 2025). This situation can easily lead to negative emotions, including anxiety and depression, which hinder the ability to perceive bodily signals accurately. Patients may become preoccupied with their symptoms or uncertain about their future health. Furthermore, physical symptoms such as dyspepsia and edema can directly impair a patient’s ability to concentrate, making it difficult for them to maintain a clear awareness of physiological signals in non-medical settings (Fang et al., 2025).

The self-regulation score (3.64 ± 0.58) and the trust score (3.62 ± 0.51) were the highest, indicating that patients demonstrated a strong ability for self-management and a high level of trust in the medical system during disease management. This may be due to the patients’ long-term adaptation to their condition, a strong social support network, and positive experiences with high-quality medical services (Ramalho and de Albuquerque, 2024).

Combined with the analysis of the environment of county hospitals, it has been observed that there are significant differences between county hospitals and city hospitals regarding medical resources, technical support, and patient education (Cui et al., 2025). To address these disparities, some localities have implemented hierarchical diagnosis and treatment systems, promoted the high-quality development of public hospitals, and enhanced the construction of county-level traditional Chinese medicine hospitals. These initiatives aim to improve the medical service capacity of county-level hospitals (Tu et al., 2024).

Managers are encouraged to enhance patient stratification education and symptom monitoring, promote the development of psychological support networks, optimize medical service processes and resource allocation, facilitate policy coordination, and empower patients to take an active role in their care. These efforts aim to help patients improve their self-awareness and perception, enabling them to manage their conditions better and enhance their quality of life.

### CHF patients living in cities/counties had a relatively high level of intrinsic perception

In this study, 406 patients with chronic heart failure from county hospitals were analyzed. It was found that 35.8% (158 patients) of urban/county patients had a mean score of 111.68 ± 16.99, which was significantly higher than the mean score of 106.48 ± 14.95 for those in rural areas. The primary factors contributing to this difference include enhanced access to technology and improved clinician-patient communication, supported by integrated healthcare systems in county hospitals. The integrity of symptom monitoring data is bolstered through the joint outpatient clinic model between county hospitals and higher level hospitals (Whitfield et al., 2025). Additionally, the quality of doctor–patient communication is superior, with longer consultation times and a lower rate of misunderstanding regarding doctors’ orders (Fang et al., 2025).

However, due to the fragmentation of resources, the lack of formalized social support, and limited health literacy, rural patients often exhibit a low level of internal perception. In the future, it is essential to enhance the internal perception capabilities of rural patients through a strategy that combines “resource allocation, community empowerment, and technology integration,” a linkage system connecting experts from higher level hospitals, county hospitals, and community health services to develop specialized health applications. Simultaneously, we should leverage the strengths of urban patients and explore the “AI early warning and family empowerment” model to improve adherence to symptom monitoring.

### The lower the number of hospitalizations for heart failure in CHF patients in the past year, the higher the level of internal perception

The results of the regression analysis indicated that the number of hospitalizations for heart failure in patients with CHF over the past year was significantly negatively correlated with the level of intrinsic perception. This finding suggests that a lower number of hospitalizations is associated with a higher level of inherent perception. This conclusion not only highlights the intrinsic relationship between disease stability and the ability to perceive internal signals but also offers important insights for clinical practice. A reduction in hospitalizations reflects better control of the patient’s condition, improved stability of the physiological system, and heightened sensitivity in the body’s internal signal transduction, allowing patients to perceive changes in physiological indicators (Ramalho and de Albuquerque, 2024) more accurately.

At the same time, maintaining stable treatment continuity helps patients establish a regular self-monitoring routine and enhances their ability to predict disease progression (Urban et al., 2024). Additionally, reducing psychological dependence on medical interventions fosters the development of self-perception skills. Therefore, clinical interventions should prioritize minimizing hospitalization frequency by optimizing pharmacotherapy and reinforcing lifestyle modifications. These strategies not only improve patient prognosis but also enhance self-management capabilities by elevating the level of internal awareness. Furthermore, the allocation of medical resources should be optimized, and out-of-hospital management should be strengthened for high-risk patients who frequently require hospitalization. Educating patients to participate in disease management actively is crucial for breaking the vicious cycle of “hospitalization–deterioration–rehospitalization” and improving both the level of internal awareness and the quality of life for patients (Sun et al., 2025).

### Patients with CHF with normal BMI had higher levels of internal perception

The paradoxical relationship between BMI and internal perception—initially indicated by higher MAIA-2C scores in individuals with high BMI but later contradicted by a negative multivariate regression coefficient—can be elucidated through confounding and non-linear mechanisms. First, the univariate association likely reflects confounding by psycho social factors: individuals in higher BMI groups exhibited stronger social support and lower levels of depression, both of which are known to enhance self-monitoring behaviors and may temporarily mitigate the metabolic burden of obesity. However, after adjusting for these confounders, the independent detrimental effect of BMI became apparent, particularly within the obese subgroup, suggesting a threshold effect where neuroendocrine regularization disrupts interoceptive signaling. Furthermore, the significant quadratic term revealed a U-shaped relationship, indicating dual pathways: malnutrition-induced somatic inattention in underweight patients and chronic inflammation-driven sensory impairment in those with obesity (Rubino et al., 2025). Clinically, these findings advocate for stratified interventions—prioritizing metabolic management for obese patients while addressing psycho social barriers in individuals with normal BMI (Rubino et al., 2025). Future studies should investigate the synergies between mindfulness-based therapies and metabolic interventions to optimize interoceptive outcomes in populations with CHF.

### Depression is a negative predictor of intrinsic perception

This study identified a significant negative correlation between depression scores and internal perception, influenced by microbiological, behavioral, and psycho social mechanisms. Dysfunction in the prefrontal cortex associated with depression disrupts attention to physiological signals, impairing non-distraction—the ability to avoid distraction and worry (Jiang H. et al., 2025). Symptom overlap further complicates bodily awareness, while low self-efficacy in patients with depression diminishes adherence to self-care, exacerbating interoceptive deficits (Liu et al., 2024). Clinically, incorporating depression management into the care of patients with CHF may help restore interoceptive function by targeting PFC activity and alleviating somatic symptom burden. Future research should prioritize early screening for depression and implement psychological interventions to optimize patient outcomes.

### The higher the level of social support, the stronger the internal perception

The results of this study indicated that social support has a positive influence on the internal perception of patients with CHF. Patients who reported high levels of social support demonstrated a greater internal locus of control. Social support encompasses various forms of assistance, including spousal support, support from children, workplace communication and achievements, social status, and respect. Adequate social support is crucial for both physical and mental health, as it helps alleviate stress related to illness and fosters recovery (Cai et al., 2025). Studies have shown that increasing the level of social support can enhance self-management abilities, improve quality of life, and reduce the readmission rate of patients with CHF (Thankachen et al., 2025). Emotional support from family members, combined with professional guidance from healthcare providers, can help patients develop a proper framework for recognizing symptoms and improve their self-monitoring skills. It is recommended that a three-dimensional support system comprising “patient–family–medical care” be established in clinical practice. This multidimensional support network can not only elevate the level of internal perception but also promote overall prognosis improvement by enhancing the quality of family support, establishing mutual support groups among patients, and employing motivational interviewing techniques.

### The lighter the symptom burden, the stronger the patient’s internal perception

The results of this study demonstrated that symptom burden has a significantly negative impact on the intrinsic perception of patients with CHF. Specifically, as symptom burden increases, the level of inherent perception decreases. Research has indicated that severe symptoms consume substantial cognitive resources, which diminishes patients’ ability to focus on physiological signals and reduces their sensitivity to physical changes (Wu et al., 2025). Complex combinations of symptoms, particularly those associated with atrial fibrillation, can make it challenging for patients to differentiate between the origins of various symptoms. This can lead to symptom confusion and diminish the accuracy of symptom reporting (Trentini and Dan-Glauser, 2025). The results indicate that it is essential to integrate symptom management into strategies aimed at enhancing internal perception abilities. This includes quantifying symptom burden using multidimensional assessment tools, developing personalized symptom control plans, and implementing training to improve symptom perception. These measures can help patients reconstruct their understanding of the relationship between symptoms and physiological responses, ultimately breaking the vicious cycle of symptom burden and declining internal perception (Maguire et al., 2021). Our findings support the development of interoception-targeted interventions for patients with CHF, including biofeedback-assisted mindfulness training to calibrate bodily awareness through real-time monitoring of signals; symptom guides to help patients distinguish between depressive symptoms and CHF exacerbations, and digital phenotyping and wearable sensors that integrate EMA for continuous interoceptive tracking. Such innovations could fill the gap between interoceptive awareness research and precision self-management.

### Cultural contextualization

While our findings reflect the county-level healthcare context in China, where disparities in resources between rural and urban areas, along with familial support norms, may amplify interoceptive differences, similar mechanisms are likely to operate across cultures. We advocate for replication studies in diverse healthcare systems to examine how systemic factors influence these pathways.

### Limitations

First, the cross-sectional design limits the ability to make causal inference. Second, convenience sampling may introduce selection bias. Third, the MAIA-2C scale requires further validation in populations with CHF.

## Conclusion

This study found that the internal perception ability of patients with CHF was influenced by multiple factors, including residence, the number of hospitalizations for heart failure in the past year, BMI, depression, social support, and symptom burden. Based on these findings, it is recommended that health education be enhanced for rural patients, transitional care be implemented for those with recurrent hospitalizations, coronary therapy be optimized for patients with ischemic cardiomyopathy, weight reduction be promoted for obese patients, psychological interventions be provided for those experiencing depression, social support be improved, and symptom burden be monitored. The sample for this study was limited to six hospitals in Nantong city and county, which may introduce selection bias. Future studies should aim to expand the sample size, adopt a high-quality cohort study design, explore additional potential influencing factors, and track long-term changes in patients’ intrinsic perception. This will allow for a deeper investigation into the ongoing impact of changes in inherent perception on patients’ disease symptoms and quality of life, ultimately providing a foundation for developing more effective nursing strategies.

## Data Availability

The raw data supporting the conclusions of this article will be made available by the authors, without undue reservation. This study has been approved by the Ethics Committee of Rudong County People’s Hospital (No. 54 [2024]), and all respondents have informed consent and voluntary participation.
